# Supplementary material for: Strategies for Mitigating Commercial Sensor Chip Variability with Experimental Design Controls
Source: Sensors (Basel). 2023 Jul 26;23(15):6703. doi: 10.3390/s23156703 (PMC10422579; doi:10.3390/s23156703)
Supplement: Supplementary file 1 [file sensors-23-06703-s001.zip › sensors-2501058-supplementary.pdf]

Supplementary Materials

SI Table S1: Sequences of tandem repeat proteins utilized in these experiments.

SI Figure S1: Comparison of immobilization and binding signals on the same chip versus different chips for R58.

SI Figure S2: Measured glass thickness across a set of blank chips.

References

Repeat	Sequence	Coverage
**R2**	AAGPLLMPFTLNFTITNLQYEEDMRRTGSRKFNTMESVLQGLLKPLFKNTSVGPLYSGCRLTLLRPEKDGAATGVDAIC<br>THRLDPKSPGLNREQLYWELSKLTNDIEELGPYTLDRNSLYVNGFTHQSSVSTTSTPGTSTVDLRTSGTPSSLSSPTIM	100%
**R5**	AAGPLLVPFTLNFTITNLQYEEDMHHPGSRKFNTTERVLQGLLGPMFKNTSVGLLYSGCRLTLLRSEKDGAATGVDAIC<br>THRLDPKSPGVDREQLYWELSQLTNGIKELGPYTLDRNSLYVNGFTHQTSAPNTSTPGTSTVDLGTSGTPSSLPSPT	100%
**R6**	SAGPLLVPFTLNFTITNLQYEEDMRHPGSRKFNTTERVLQGLLKPLFKSTSVGPLYSGCRLTLLRSEKDGAATGVDAIC<br>THRLDPKSPGVDREQLYWELSQLTNGIKELGPYTLDRNSLYVNGFTHQTSAPNTSTPGTSTVDLGTSGTPSSLPSPT	67%
**R7**	SAGPLLVPFTLNFTITNLQYEEDMHHPGSRKFNTTERVLQGLLGPMFKNTSVGLLYSGCRLTLLRPEDNGAATGMDAIC<br>SHRLDPKSPGLNREQLYWELSQLTHGIKELGPYTLDRNSLYVNGFTHRSSVAPTSTPGTSTVDLGTSGTPSSLPSPT	100%
**R9**	TAGPLLVPFTLNFTITNLQYEEDMHRPGSRKFNATERVLQGLLSPIFKNSSVGPLYSGCRLTSLRPEKDGAATGMDAVC<br>LYHPNPKRPGLDREQLYWELSQLTHNITEGLPYSLDRDSLYVNGFTHQNSVPTTSTPGTSTVYWATTGTPSSFPGHT	96%
**R11**	EPGPLLIPFTFNFTITNLHYEENMQHPGSRKFNTTERVLQGLLKPLFKNTSVGPLYSGCRLTLLRPEKHEAATGVDTIC<br>THRVDPIGPGLDRERLYWELSQLTNSITELGPYTLDRDSLYVNGFNPRSSVPTTSTPGTSTVHLATSGTPSSLPGHT	100%
**R25**	TAGPLLVPFTLNFTITNLKYEEDMHCPGSRKFNTTERVLQSLLGPMFKNTSVGPLYSGCRLTLLRSEKDGAATGVDAIC<br>THRLDPKSPGVDREQLYWEKSQLTNGIKELGPYTLDRNSLYVNGFTHQTSAPNTSTPGTSTVDLGTSGTPSSLPSPT	97%
**R34**	APVPLLIPFTLNFTITDLHYEENMQHPGSRKFNTTERVLQGLLKPLFKSTSVGPLYSGCRLTLLRPEKHGAATGVDAIC<br>TLRLDPTGPGLDRERLYWELSQLTNSITELGPYTLDRDSLYVNGFNPWSSVPTTSTPGTSTVHLATSGTPSSLPGHT	100%
**R58**	ATGPVLLPFTLNFTITNLQYEEDMHRPGSRKFNTTERVLQGLLMPLFKNTSVSSLYSGCRLTLLRPEKDGAATRVDAVC<br>THRPDPKSPGLDRERLYWKLSQLTHGITELGPYTLDRHSLYVNGFTHQSSMTTTRTPDTSTMHLATSRTPASLSGPT	95%

SI Table S1: Sequences of tandem repeat proteins utilized in these experiments. *The sequences and numbering scheme were sourced from the published sequence [1]. Protein expression was confirmed via LC/MS-MS as described in our previous work [2] and coverage is reported in the table.*

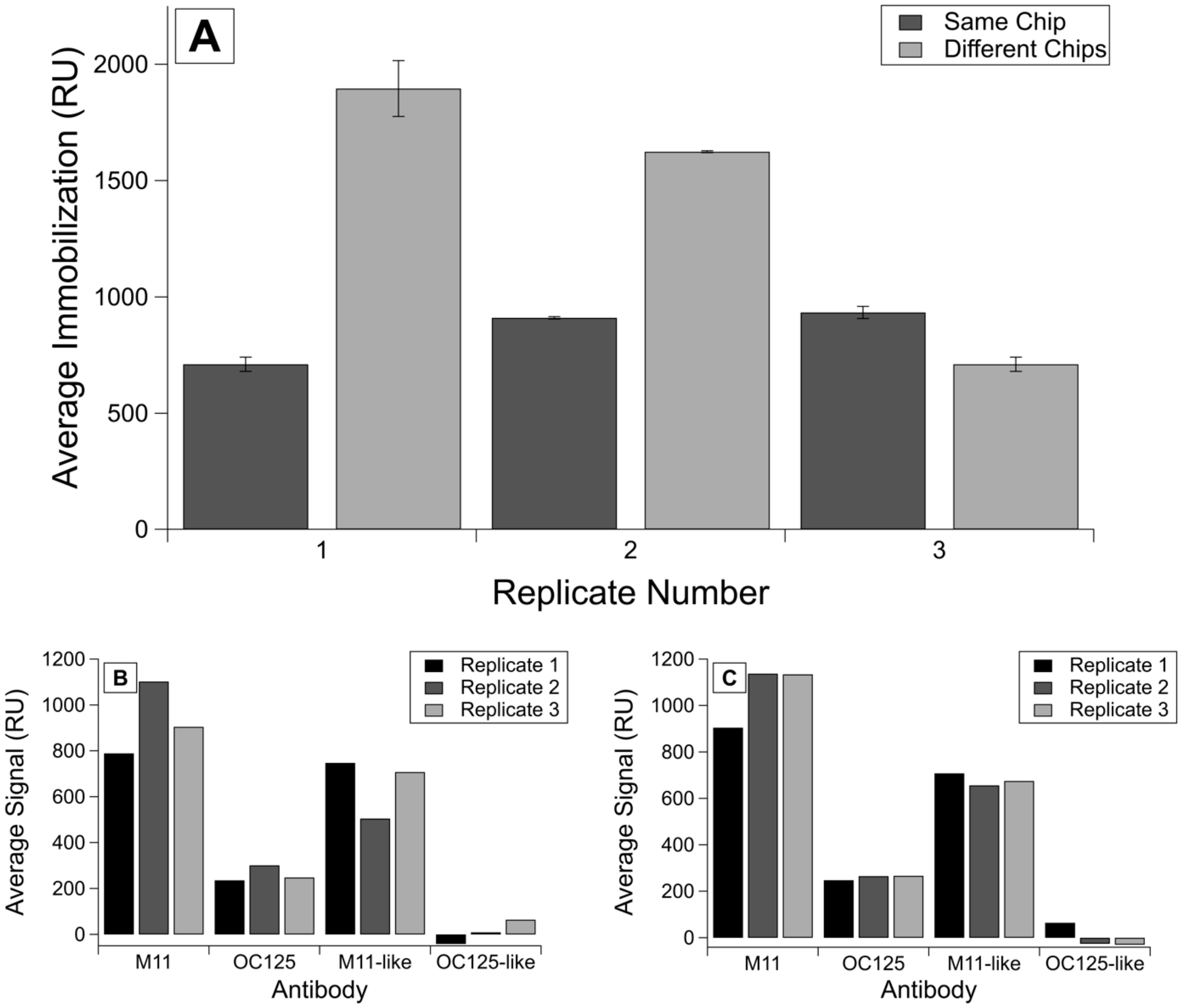

SI Figure S1: Comparison of immobilization and binding signals on the same chip versus different chips for R58. *Shown is A) the averaged corrected signal for each immobilization of R58 protein per replicate on the same chip or on different chips. Error bars are standard error of the mean, with n=4. Also shown are averaged corrected signal for each antibody injection by replicates shown in B) the analyte binding on different chips and C) the analyte binding on the same chip.*

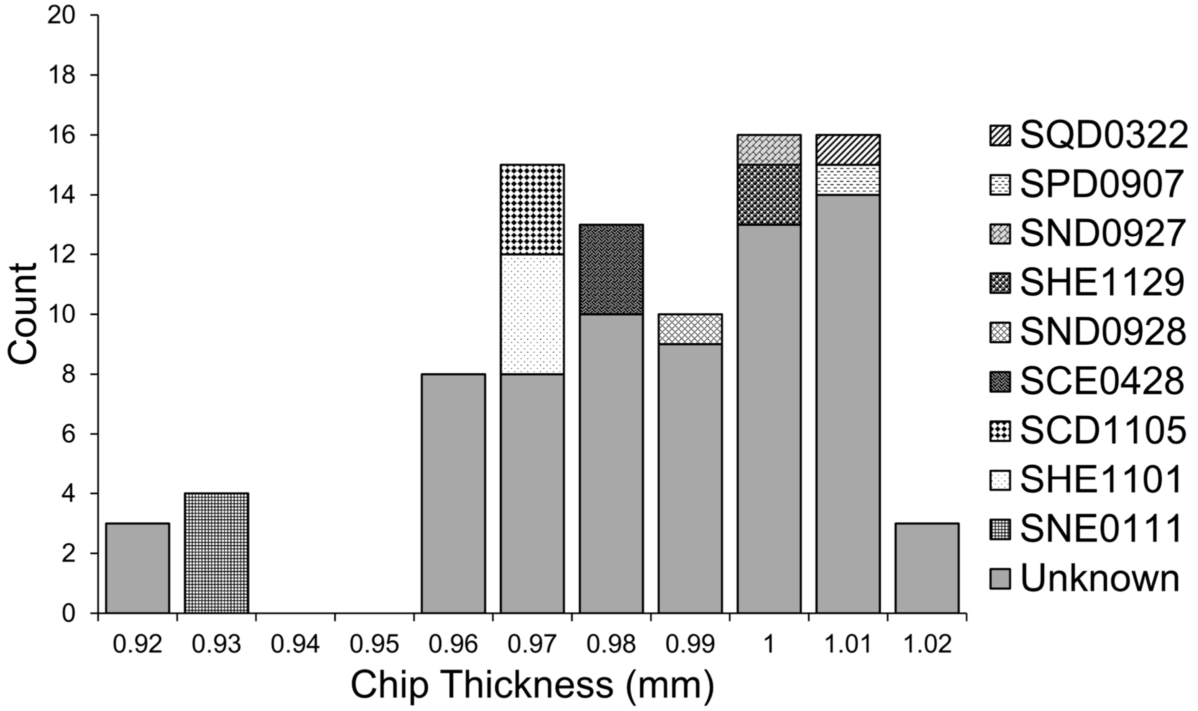

SI Figure S2: Measured glass thickness across a set of blank chips. *Utilizing digital calipers, 88 blank chips were measured. Twenty of the chips were from known sensor lots (lot numbers given in legend with corresponding colors), and other chips were from unknown lots.*

References

1. O'Brien, T. J.; Beard, J. B.; Underwood, L. J.; Dennis, R. A.; Santin, A. D.; York, L., The CA 125 Gene: An Extracellular Superstructure Dominated by Repeat Sequences. *Tumor Biology* **2001**, 22, (6), 348-366.

2. Wang, C.-W.; Hanson, E.K.; Minkoff, L.; Whelan, R. J., Individual recombinant repeats of MUC16 display variable binding to CA125 antibodies. *Cancer Biomarkers* **2023**, 37, (2), 85-94.
